# Layer-by-Layer Biopolymer-Coated Deformable Liposomes–In Situ Gel: A Hybrid Strategy for Enhanced Ocular Delivery of Itraconazole: In Vitro and In Vivo Appraisal

**DOI:** 10.3390/gels11010019

**Published:** 2024-12-31

**Authors:** Mohamed M. Badran, Areej Alsubaie, Mounir M. Salem Bekhit, Abdullah H. Alomrani, Aliyah Almomen

**Affiliations:** 1 Department of Pharmaceutics, College of Pharmacy, King Saud University, Riyadh 11451, Saudi Arabia; 439204066@student.ksu.edu.sa (A.A.); mbekhet@ksu.edu.sa (M.M.S.B.); aomrani@ksu.edu.sa (A.H.A.); 2 Nanobiotechnology Research Unit, College of Pharmacy, King Saud University, P.O. Box 2457, Riyadh 11495, Saudi Arabia; 3 Department of Pharmaceutical Chemistry, College of Pharmacy, King Saud University, Riyadh 11495, Saudi Arabia; alalmomen@ksu.edu.sa

**Keywords:** Itraconazole, deformable liposomes, coating, in situ gel, cornea, antifungal efficacy

## Abstract

Itraconazole (ITZ) is a potent antifungal agent. Its oral administration is associated with systemic toxicity, and its efficacy in ocular formulations is limited. This study aims to enhance ITZ’s ocular permeation and antifungal efficacy by loading it into deformable liposomes (DLs) based on Tween 80 (T) or Poloxamer 188 (P). Moreover, ITZ was loaded into biopolymer-coated DLs to augment its ocular availability. ITZ-loaded DLs were coated with hyaluronic acid (HA-DLs), chitosan (CS-DLs), or a layer-by-layer coating (CS/HA-DLs). These formulations were further laden into pH-sensitive in situ gels to provide a hybrid system to intensify their ocular adhesion properties. The prepared DLs were successfully prepared with vesicle sizes in nonorange (<200 nm). The zeta potential values of DLS were negative before coating and shifted to high negativity with HA coating and positivity with CS and CS/HA bilayer coating. These variations of zeta potential indicate successful CS and HA coatings. The optimized A high EE% was achieved with DLs-T: 89% (CS/HA-DLs-T), 86% (CS-DLs-T), 85% (HA-DLs-T), and 79% (HA-DLs-T). Therefore, DLs-T were incorporated into in situ gels, displaying optimal gelling capacity and viscosity. The release rate of ITZ from the coated DLs-laden in situ gels was slower than that observed with the uncoated DLs-gel. CS/HA-DLs-T laden-in situ gels showed the highest ex vivo transcorneal permeability and antifungal efficacy. These data suggest that the layer-by-layer-CS/HA-DLs-T presents a hopeful strategy for the ocular delivery of ITZ, offering a promising approach for managing ocular fungal infections.

## 1. Introduction

Ocular drug delivery encounters significant challenges, mainly due to rapid drug clearance caused by nasolacrimal drainage and several ocular barriers. The lipid-rich epithelium and endothelium restrict the permeability of hydrophilic drugs, while the stroma presents a barrier to hydrophobic medications [1]. These factors limit the bioavailability of therapeutic agents, especially in treating fungal eye infections [2]. Normal eye drops fail to deliver an acceptable drug concentration to the targeted ocular tissue when treating fungal infections. The rising incidence of ocular fungal infections was noticed, which may result in severe complications such as vision loss [3,4].

Itraconazole (ITZ) is an effective antifungal agent. It has a very low aqueous solubility and is classified as a BCS class II [5]. The high lipophilicity of ITZ (logp = 6.2) and considerable molecular weight (705.6 g/mol) might reduce its permeation after ocular administration. These properties can hinder ITZ ocular availability and decrease its effectiveness in treating ocular fungal infections [5]. For instance, Mochizuki et al. evaluated the penetration of ITZ into the aqueous humor and vitreous after oral administration of ITZ capsules (200 mg/day) over 12 days for a 21-year-old male to treat fungal endophthalmitis [6]. The concentrations of ITZ were 0.492 µg/mL in the plasma, 0.020 µg/mL in the vitreous, and none in the aqueous humor. The fungal infections were absent in the plasma; however, fungal DNA was detected in the vitreous. The study’s outcomes suggested oral ITZ is limited in ocular tissue [6]. Delivering drugs effectively to ocular tissues for treating fungal infections is challenging. Various approaches have been established to increase the ocular permeability and retention of the drugs. Liposomes have shown a promising approach in ophthalmic applications due to their biocompatibility and biodegradability [7]. Deformable liposomes (DLs) are more elastic than conventional liposomes. DL elasticity plays a vital role in drug permeation through biological barriers like the cornea [7,8]. Edge activators such as hydrophilic surfactants (e.g., Tween 80; TW80) were found to increase the elasticity of the liposomal membrane due to their interference with the phospholipid bilayer that forms the liposomal membrane [9]. Furthermore, the ocular delivery of the drug-loaded DLs could be augmented by covering their outer surface with biopolymers of chitosan (CS), hyaluronic acid (HA), or a combination. The electrostatic interaction between CS and the mucin layer on the cornea can improve the residence time of the drug-loaded carriers, which facilitates drug permeation [1]. CS exhibits high mucoadhesive properties with antibacterial and antifungal activities [9]. HA is a naturally occurring polysaccharide found in the extracellular matrix of ocular tissues. HA has gained significant attention for its improved intraocular permeation and prolonged retention times, making it an ideal excipient for ocular applications [10]. It can promote the hydration of the ocular surface, producing non-irritant formulations [11]. Therefore, this work focuses on formulating ITZ-DLs and explores the impact of surface-modified materials such as HA and CS on DL properties. These coated carriers are expected to improve the ocular delivery of ITZ. In addition, incorporating these biopolymer-coated DLs into an in situ gel system could further prolong the retention time and boost the antifungal efficacy of ITZ.

## 2. Results and Discussion

The present study explored the potential application of ITZ-loaded DLs for ocular delivery and antifungal activity [12]. Surface-modified DLs could improve the bioadhesive properties of DLs, extend ocular residence time, and increase the ocular availability of entrapped ITZ [13]. Concurrently, incorporating the surface-modified DLs into an in situ gel system can further augment the pharmacological activity of the drugs, offering a promising strategy for ophthalmic application [13]. Therefore, this study evaluates the impact of surfactant ratios and coating layers on optimizing DL characteristics, aiming to develop an effective ocular carrier for ITZ.

**Figure 1:**
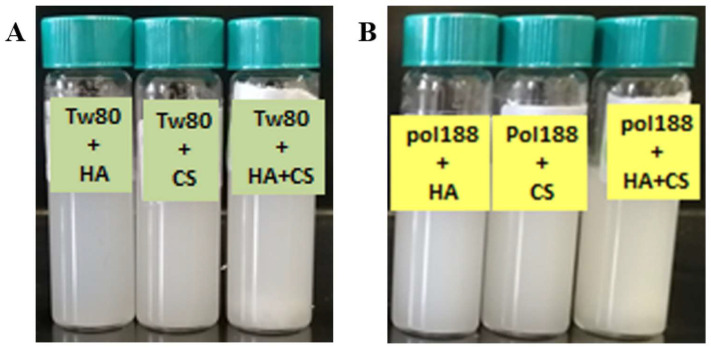
Biopolymers-coated deformable liposomes (DLs) formulated with TW80 (**A**) and PL188 (**B**), hyaluronic acid (HA), chitosan (CS), and their combination (CS/HA).

DLs were prepared using soybean phospholipids and Chol combined with TW80 or PL188 as hydrophilic surfactants. Initially, proliposomes were successfully formulated using the coacervation phase separation method [14], yielding a uniform, semisolid gel with a yellowish-brown color (Figure 1). After adding 10 mL of PBS (pH 7.4), 2% HA, and/or 0.5% CS, the proliposomes transformed into liposomal dispersions (Figure 1). The DLs containing TW80 (DLs-T) were surface-modified with HA, CS, and a layer-by-layer of HA and CS to produce HA-DLs-T, CS-DLs-T, and CS/HA-DLs-T, respectively. Similarly, DLs containing PL188 (DLs-P) were surface-modified with the same coating polymers to obtain HA-DLs-P, CS-DLs-P, and CS/HA-DLs-P.

### 2.1. Vesicle Size Distribution and Zeta Potential

Different DLs were formulated using two types of surfactants (TW80 and PL188) and varying ratios and were further surface-coated with biopolymers. The obtained formulations were qualified by measuring vesicle size, PDI, and zeta potential.

Firstly, using the ratios of 0.9%, 0.1%, and 0.1% of SPC, TW80 or PL188, and Chol, respectively, DL-T (TW80) exhibited a small vesicle size of 79.8 ± 4.5 nm. Upon surface modification, the vesicle sizes increased to 97.9 ± 3.7 nm for HA-DLs-T, 121.2 ± 5.4 nm for CS-DLs-T, and 161.1 ± 6.8 nm for CS/HA-DLs-T, respectively. Similarly, for DLs containing PL188 (DLs-P), the vesicle size was 67.7 ± 3.2 nm, which increased after surface modification to 86.6 ± 3.8, 101.8 ± 4.5, and 175.9 ± 3.1 nm for HA-DLs-P, CS-DLs-P, and CS/HA-DLs-P, respectively. Uncoated DLs-T and DLs-P (PL188) showed slight negative charges of −2 mV and −5 mV, respectively. The addition of HA increased the negative charges, resulting in zeta potential values of −12 mV and −18 mV for HA-DLs-T and HA-DLs-P, respectively. Conversely, CS-DLs-T and CS-DLs-P showed positively charged vesicles with zeta potential values of 30 mV to 35 mV, respectively. The CS/HA-DLs-T and CS/HA-DLs-P displayed 25 and 16 mV zeta potential values, respectively.

On the other hand, increasing the amount of surfactant, as in 0.9% SPC, 0.9% TW80 or PL188, and 0.1% Chol, resulted in a further decrease in the vesicle sizes. DL-T (W80) exhibited the smallest vesicle size of 55.45 ± 0.38 nm, which increased after surface modification to 62.6 ± 1.3, 70.1 ± 0.5, and 81.5 ± 0.3 nm for HA-DLs-T, CS-DLs-T, and CS/HA-DLs-T, respectively. By using PL188y, the vesicle size decreased to 44.69 ± 0.5 nm, 59.7 ± 0.4, 67.8 ± 0.3, and 75.9 ± 3.1 for DLs-P, HA-DLs-P, CS-DLs-P, and CS/HA-DLs-P, respectively. Zeta potential values of DLs-T and DLs-P (PL188) were lower negative charges of −4 mV and −7 mV, respectively. After surface modification with HA, zeta potential values increased by −19 mV and −21 mV for HA-DLs-T and HA-DLs-P, respectively. CS-DLs-T and CS-DLs-P displayed high positive charges of 31 mV to 40 mV, respectively. CS/HA-DLs-T and CS/HA-DLs-P exhibited zeta potential values of 30.5 and 5 mV, respectively.

There is a clear correlation between surfactant type and concentration on the vesicle size and zeta potential values. The surface coating led to predictable increases in the vesicle size. The magnitude of the vesicle size values confirmed the effective surface modification of DLs by using HA, CS, and CS/HA, which could improve the potential functionality of DLs. Moreover, the variation in vesicle size in DLs containing TW80 and PL188 could be attributed to their diverse surface activities and chemical structures. PL188, with its relatively high HLB (29), helps the formation of a tightly packed liposome structure, leading to smaller vesicle sizes [15]. Also, it could create a protective hydrophilic layer around DLs that prevents vesicle aggregation, resulting in a smaller size. In comparison, TW80, characterized by a lower HLB (15), leads to the formation of larger vesicles due to its bulkier structure [16]. The zeta potential value of the particles is an important property that could affect the physical stability of the colloidal system [17]. This property leads to less efficient interaction with lipids, contributing to the formation of larger vesicle sizes. A high zeta potential value of the particle, whether negative or positive, usually prevents aggregation due to a strong electric repulsion force. Conversely, particles with low zeta potential values are prone to aggregate easily and deteriorate the stability of the colloidal systems [17]. Therefore, surface modifiers could increase the physical stability of colloidal particles by electrostatic and/or steric stability [18].

PDI is a key parameter for assessing the homogeneity of the particle size distribution, with a smaller PDI value indicating a narrower size distribution [19]. In the current study, DLs with TW80 exhibited PDI values ranging from 0.245 to 0.331, reflecting a relatively homogenous vesicle size distribution. In contrast, DLs containing PL188 displayed PDI values between 0.311 and 0.708, indicating a broader size distribution. These data suggest that the surface modification could enhance the homogeneity of DLs with TW80, while PL188 had an unfavorable effect on DL size uniformity. This effect of PL188 indicates that the liposomes are not well-formed [20].

### 2.2. Entrapment Efficiency of ITZ-Loaded Biopolymer-Coated DLs

The EE% is one of the most crucial factors in determining the success of nanocarrier systems to improve the ocular availability of the drug. It has been reported that edge activators significantly increased the EE% of the drugs in DLs compared to conventional liposomes. The values of EE% of DLs containing TW80- or PL188-based DLs before and after surface modification.

Based on the ratio of 0.9% SPC, 0.1% TW80, or PL188, and 0.1% Chol, the EE% of DLs-T, HA-DLs-T, CS-DLs-T, and CS/HA-DLs-T was 65%, 74%, 78.5%, and 83.8%, respectively. In the case of PL188, the lower EE% of ITZ was observed as 52%, 62%, 58%, and 65% for DLs-P, HA-DLs-P, CS-DLs-P, and CS/HA-DLs-P, respectively. But by using the ratio of 0.9% SPC, 0.9% TW80, or 0PL188, and 0.1% Chol, the EE% was increased for DLs formed by W80 to be 79%, 85%, 86%, and 89% for DLs-T, HA-DLs-T, CS-DLs-T, and CS/HA-DLs-T, respectively. While DLs using PL188 exhibited the lowest values of EE% to 35%, 37%, 46%, and 40% for DLs-P, HA-DLs-P, CS-DLs-P, and CS/HA-DLs-P, respectively. DLs prepared with PL188 exhibited a notable amount of precipitate formed. The DLS prepared with TW80 exhibited the highest EE% for several reasons. They include the moderate HB (15), which stabilizes the DL bilayer effectively and enables strong interactions with lipophilic drugs, promoting better entrapment of the drug [21,22,23]. The low EE% of PL188-based DLS may be due to its hydrophilic nature (HLB 29), which may not be enough to support liposomal structure [24]. The low EE% of PL188-based colloidal systems has been reported due to its limited hydrophobicity compared to the other surfactants [25]. The low hydrophobicity leads to weak or few hydrophobic interactions between the surfactant molecules and the drug, reducing the value of EE%. A minimal amount of ITZ was entrapped in the formulation containing a high concentration of PL88. These results may be attributed to phase separation, precipitation, or micelle formation, which might expel the ITZ from the formulation [26]. EE% was increased in the presence of surface coating such as HA and CS, which may be attributed to the effect of additional interactions [27,28]. Therefore, selecting surfactant, coated polymer, and component ratio is important to improve drug loading in liposomal formulations. Based on the minimum vesicle size and maximum EE% results, the formulations with a ratio of 0.9% SPC, 0.9% TW80, and 0.1% Chol were selected as the optimal composition for continuous experiments.

### 2.3. Morphology of ITZ-Loaded Biopolymer-Coated DLs

**Figure 2:**
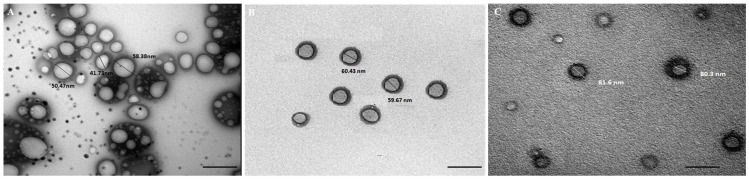
Transmission electron microscopy of DLs-T (**A**), CS-DLs-T (**B**), and CS/HA-DLs-T (**C**) (bar = 100 nm).

The formed CS-DLs-T and CS/HA-DLs-T morphology was visualized using TEM. These formulations were selected due to their highest EE% values. DLs-T was also selected for comparison issues. The TEM pictures prove that liposomes were created with a spherical shape (Figure 2). The TEM pictures also showed a thick layer around CS-DLs-T and CS/HA-DLs-T, confirming surface modifiers on DLs. The biopolymer coating impacted the surface characteristics of the DLs in terms of preserving their spherical shape. The smaller sizes observed in the formulation using TEM, compared to a Zetasizer Nano ZS, can be attributed to the different states of the vesicles during measurement. TEM was performed on vesicles in their dehydrated state, leading to small sizes, whereas a Zetasizer Nano ZS measured the vesicles in their hydrated state, reflecting hydrodynamic diameter.

### 2.4. Stability Study

The variations in vesicle size and zeta potential values for the liposomal dispersions were monitored after one month (Figures S1 and S2). A slight change in the vesicle size and zeta potential was detected at 4 °C (*p* < 0.05), but still, it was within the adequate range. This effect can be ascribed to liposomal dispersions’ lower mobility and permeability [9]. The coated liposomes with HA or CS showed a minor increase in size due to the occurrence of the coating layers. Similarly, in a study by Badran et al.), the stability of CS-coated metoprolol liposomes was examined [7]. The CS-coated layer over the liposomes was expected to preserve the physical stability of the vesicles, further confirming the role of coating in improving stability.

### 2.5. Characterization of ITZ-Loaded Biopolymer-Coated DLs–In Situ Gel

One of the significant challenges with liquid ophthalmic formulations is their rapid clearance from the ocular surface, primarily due to tear turnover and frequent blinking [29]. Increasing viscosity and mucoadhesion properties are commonly employed strategies to prolong the residence time at the application site [30]. In situ gel systems have gained considerable interest in mitigating the issue of rapid clearance. These systems undergo a sol-to-gel transformation triggered by environmental changes like pH shifts [21]. The prepared in situ gel formulations were evaluated for clarity, pH, gelling capacity, and rheological properties. Clarity was assessed by visual inspection to ensure the preparations would be comfortable for ocular use [31]. The developed in situ gel formulations demonstrated homogeneity and clarity (Table 1), indicating their potential tolerability for ophthalmic application. The pH of the in situ gel system was measured before and after mixing with STF, with the results shown in Table 1. The pH values of ITZ-G, HA-DLs-T-G, CS-DLs-T-G, and CS/HA-DLs-T-G were 4.8, 5.5, 5.5, 4.8, and 4.9, respectively. These values were in the acceptable range of physiological conditions, signifying the suitability for ophthalmic application [32]. Remarkably, the pH values of DLs-T-G and HA-DLs-T-G were slightly higher (5.5) compared to CS-DLs-T-G and CS/HA-DLs-T-G (pH 4.8–4.9), probably due to the residual acetic acid used in dissolving CS. After mixing with STF, the pH values of all formulations were increased to 6.4–6.7 (Table 1), facilitating the sol-gel transformation. The codes of gelling capacity were (+) for gels formed after a few min and dissolved quickly; (++) for immediate gelation that continued for 5–10 min; (+++) for immediate gelation that extended for longer than 2 h. The gelling capacity of CS-DLs-T-G and CS/HA-DLs-T-G was observed immediately upon contact with STF and remained stable for up to 2 h, suggesting their sustained gelation. The presence of CS could enhance the gelling capacity, as its interaction with STF promotes rapid and stable gel formation [33].

**Figure 3:**
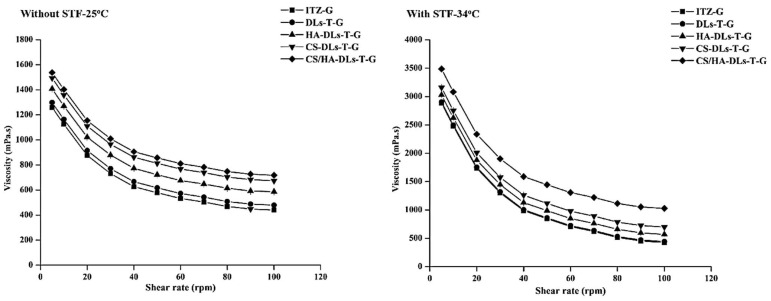
Viscosity and shear rate of ITZ-G and ITZ-loaded biopolymer-coated DLs–in situ at non-physiological conditions and physiological conditions (pH 7.4, 34 °C).

The ocular in situ *gels* should have adequate viscosity to prevent their washout from the ocular surface during blinking (20 blinks/min) [34]. Frequent blinking leads to the reduction between the formulation and the cornea or conjunctiva. The viscosity of the in situ gel has a vital role in determining its gelling capacity and the drug release profile [35]. The viscosity profile of the developed DLs–in situ gels was assessed before and after mixing with STF, as illustrated in Figure 3. The obtained formulations showed a non-Newtonian, pseudoplastic flow behavior both in the absence and presence of STF (Figure 3). Notably, the values of viscosity were decreased with an increasing shear rate. An apparent increase in viscosity was observed after mixing with STF at 35 °C, likely due to the elevated pH, indicating a successful sol-to-gel transition under physiological conditions. Furthermore, the CS-DLs-T-G and CS/HA-DLs-T-G showed higher viscosity values across all the examined shear rates than HA-DLs-T-G and ITZ-G. This viscosity behavior is helpful for ocular applications, as it can increase patient comfort and improve therapeutic efficacy [36].

### 2.6. Bioadhesion Study

**Figure 4:**
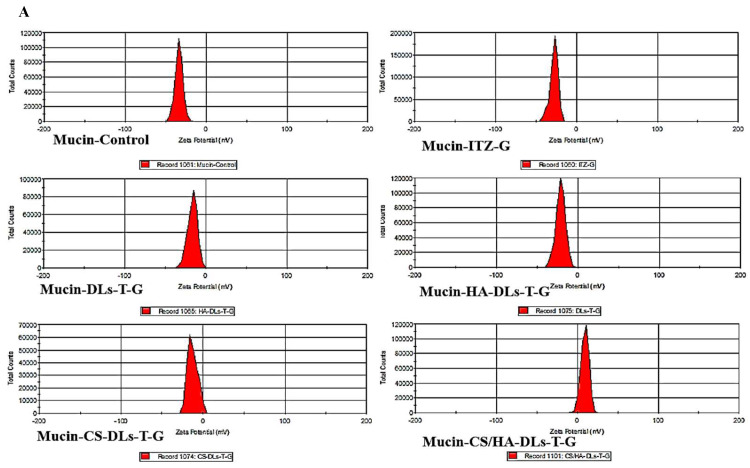

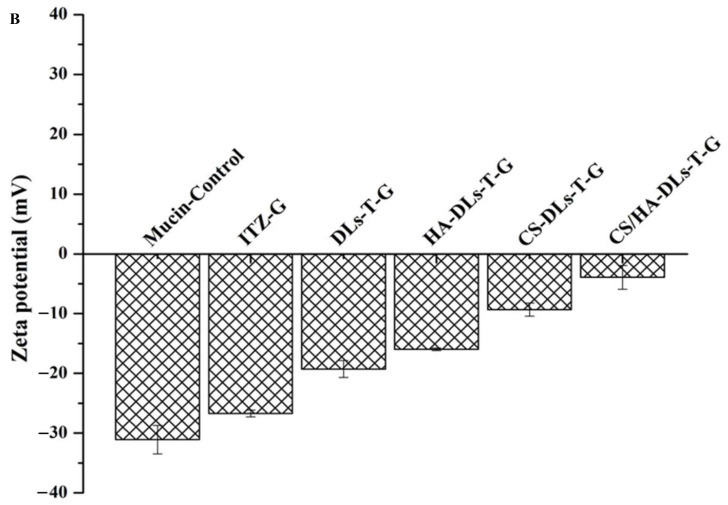
Zeta potential distribution (**A**) and histogram (**B**) of mucin solution (0.5 mg/mL), mucin-ITZ-G, mucin-DLs-T-G, mucin-HA-DLs-T-G, mucin-CS-DLs-T-G, and mucin-CS/HA-DLs-T-G. Results are expressed as means ± SD, *n* = 3.

The bioadhesion of the obtained formulations was studied by determining the potential change in surface charge after incubating with the mucin solution. The zeta potential values of the mucin solution before and after incubation were compared, and the results are depicted in Figure 4A,B. The zeta potential values were −31, −27, −19, −15, −9, and −3 mV for the mucin solution, mucin-ITZ-G, mucin-DLs-T-G, mucin-HA-DLs-T-G, mucin-CS-DLs-T-G, and mucin-CS/HA-DLs-T-G, respectively. A notable reduction in zeta potential values was observed for CS-coated formulations (*p* < 0.05). At the same time, a slight decrease was detected after incubation with ITZ-G, DLs-T-G, and HA-DLs-T-G. This effect in CS-coating can be due to the electrostatic interactions between the cationic amino groups of CS and the anionic sialic groups of mucin [7]. Additionally, HA can form hydrogen bonds with mucin, which helps create a bioadhesive network [37].

### 2.7. In Vitro Release Study

**Figure 5:**
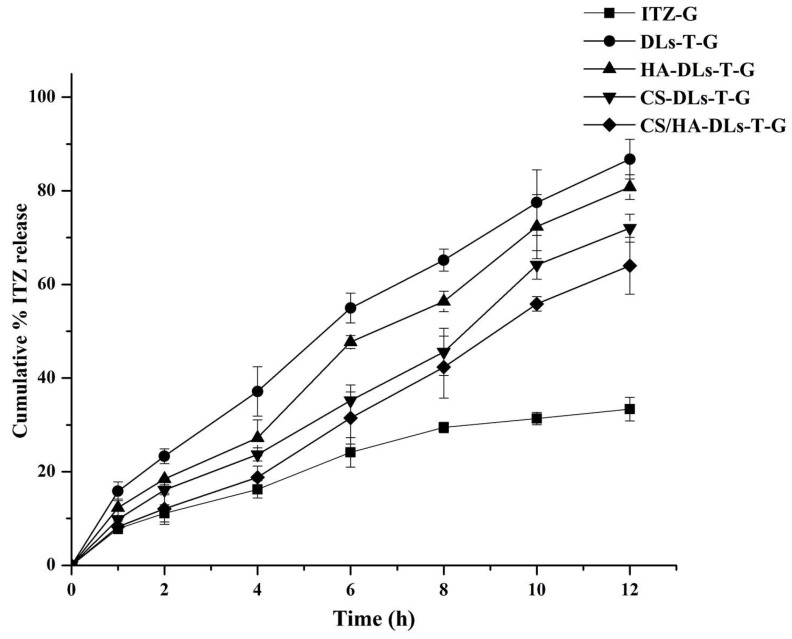
In vitro release profiles of ITZ-G and ITZ-loaded biopolymer-coated DLs–in situ gel systems.

Figure 5 illustrates the cumulative release profiles of ITZ from selected DLs-laden in situ gels in STF (pH 7.4 and 37 °C). The ITZ-G formulation achieved a rapid initial drug release of 24.13 ± 3.16% within 6 h, with a total release reaching 33.34 ± 2.52% up to 12 h. The release behavior from gel formulations is mainly governed by water penetration and the diffusion of the drug through the swelled gel network [38]. This behavior is attributable to the gel matrix, which regulates the rate of drug diffusion. The retarding effect of ITZ release from the in situ gel system is also due to its increased viscosity. The release profiles of the obtained DL formulations exhibited a biphasic pattern: an initial phase during the first 6 h, followed by slow release up to 12 h. This rapid release may be due to the presence of free ITZ in the dispersion medium and DL surface [39].

The in vitro release of ITZ from DLs-T-G showed a rapid release of 45.96 ± 3.17% at 6 h and 86.77 ± 4.23% at 12 h compared to ITZ-G (*p* < 0.05). The in vitro release of ITZ from DLs-T-G displayed a faster release compared to the free ITZ-loaded in situ gel (ITZ-G). This can be explained based on the role of TW80 (edge activator), which promotes the deformability of liposomal vesicles, facilitating a more ITZ release [7]. TW80 might lower the surface tension of liposomes, which makes it easier for drugs to move from DLs to the surrounding medium [9]. Additionally, the solubilizing property of TW80 can enhance ITZ’s localization at the DLS surface, driving the release kinetics. The smaller vesicle size of DLs further produces a larger surface area exposed to the dissolution medium, hastening the drug release [25]. Furthermore, the high value of HLB of TW80 (15) can form stable dispersions and improve the interactions between DLs and the aqueous medium [9]. These properties of TW80 could support an efficient release mechanism, thus promoting better drug delivery performance.

The release data from coated DLs–in situ gels revealed a delay of ITZ release compared to the uncoated one, probably resulting from the liposomal membrane and the coating layer [38]. The obtained coated formulations of HA-DLs-T-G, CS-DLs-T-G, and CS/HA-DLs-T-G demonstrated different release profiles after 6 h: 47.871 ± 1.35, 35.22 ± 3.34, and 31.49 ± 5.57%, respectively. The same pattern was obtained with more slow release over 12 h with 80.81 ± 2.64, 72.02 ± 2.99, and 63.96 ± 6.06%, respectively. These data suggest that the liposomal polymer coating plays a vital role in controlling drug release. The CS/HA or CS-coated layers (CS-DLs-T-G and CS/HA-DLs-T-G) are more pronounced in controlling ITZ release than the HA-coated layer (HA-DLs-T-G). The main explanation for this behavior may be the strong electrostatic interaction with positively charged CS and negatively charged DLs, creating a more compact layer [40]. HA is a more hydrophilic polymer than CS, which may permit faster hydration and diffusion of the drug [13]. In addition, the multilayered DLs (CS/HA-DLs-T-G) demonstrated the lowest amount of ITZ released after 12 h compared to other investigated formulations. Using alternate polyelectrolytes of CS and HA can achieve a denser shell structure, thereby minimizing early leakage of the entrapped drug [41]. The release kinetic order of ITZ from the tested formulations was evaluated (Table 2). The Korsmeyer–Peppas model was selected as the best kinetic release model due to its highest R^2^ value. The n values (the diffusional exponent) were observed within the range of 0.43 to 0.854. Various mechanisms govern this ITZ release profile, such as drug diffusion, relaxation, and erosion. In conclusion, CS-DLs-T-G and CS/HA-DLs-T-G depicted a slow release of ITZ compared to the uncoated one.

### 2.8. Ex Vivo Transcorneal Permeation Study

**Figure 6:**
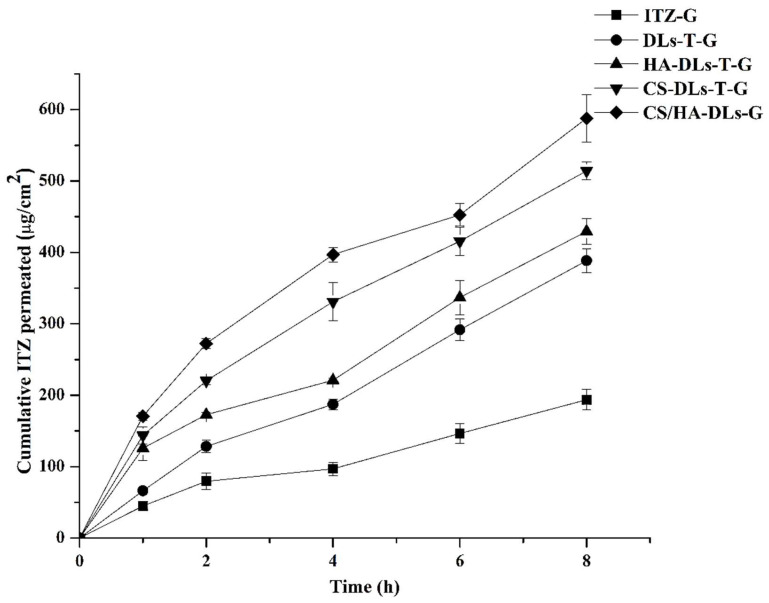
Ex vivo corneal permeation profiles of ITZ-loaded biopolymer-coated DLs–in situ gel systems (n = 3).

Figure 6 displays the cumulative permeation of ITZ through the cornea following the application of coated DLs–in situ gel formulations. As shown in Figure 6, after 8 h, ITZ permeation of ITZ was the highest for CS/HA-DLs-T-G, followed by CS-DLs-T-G, HA-DLs-T-G, and DL-T-G. While ITZ-G (control group) had the lowest corneal permeation (1.44 g/cm^2^), there was a statistically significant difference compared to the control group. ITZ-DLs-G showed a higher ITZ permeation level than ITZ-G (free drug-in situ gel). This increased permeation is due to the lipid matrix of DLs, which adheres effectively to the corneal epithelium [42]. Upon surface modification of DLs with HA and CS, the ITZ corneal permeation increased compared to uncoated DLs-T. CS/HA-DLs-T-G and CS-DLs-T-G exhibited the highest ITZ permeation levels, followed by HA-DLs-T-G, DLs-T-G, and ITZ-G. This enhanced permeation in the presence of CS can be attributed to its positive charge, which interacts electrostatically with negatively charged components in the corneal epithelial cells [33]. This interaction transiently loosens the tight junctions, facilitating the drug permeation across the corneal barrier. Furthermore, the adherence of CS formulations is strengthened by electrostatic interactions due to the anionic nature of the mucin layer on the ocular surface [43]. CS also demonstrates excellent mucoadhesive properties, offering intimate contact between CS and corneal mucosa, thus extending residence time. Accordingly, the cationic carriers attach effectively to the corneal surface, enhancing drug retention and permeation with maximum flux and permeability coefficient [44]. Tacrolimus-loaded cationic liposomes were found to prolong the precorneal time of the drug, enhance drug availability, and relieve eye dryness [44]. Moreover, HA is known for its high corneal hydration capacity due to its great water uptake properties. Thus, the CS/HA-DLs could improve the corneal permeation. Combining HA (a negative charge) with CS in one formulation could increase the hydration of the cornea, temporarily loosening the corneal barrier. However, adding multiple polymers (CS/HA-DLs-T) to the DL surface may reduce the direct interaction with the corneal surface, causing slower drug release and lower permeation [40]. However, the DLS coated with CS/HA via strong electrostatic interactions could increase DL stability with improved drug permeation compared to other formulations [40]. These data indicate that the interaction of CS with HA of the DL surface plays a crucial role in terms of ocular delivery. As listed in Table 3, the Jss of ITZ in CS/HA-DLs-T-G, CS-DLs-T-G, HA-DLs-T-G, DL-T-G, and ITZ-G were 66.13 ± 4.11^2^, 59.54 ± 1.89, 50.07 ± 1.21, 45.73 ± 1.68, and 18.84 ± 1.73 µg/(cm/h), respectively. Moreover, the values of P_eff_ were 13.22 ± 0.82 × 10^−3^, 11.91 ± 0.17 × 10^−3^, 10.02 ± 0.24 × 10^−3^, 9.15 ± 0.15 × 10^−3^, and 3.79 ± 0.35 × 10^−3^ cm/min for CS/HA-DLs-T-G, CS-DLs-T-G, HA-DLs-T-G, DL-T-G, and ITZ-G, respectively. The order lag time values were DLs-T-G < CS/HA-DLs-T-G < HA-DLs-T-G < CS-DLs-T-G < ITZ-G and ITZ-G. Compared with all formulations, ITZ formulated in CS/HA-DLs-T-G exhibited the highest values of Jss and Peff, confirming that the corneal permeation was greatly enhanced by coating layers (CS/HA) of DLs. A significant difference (*p* < 0.05) in Jss and Peff values was detected.

### 2.9. Corneal Hydration Level

The corneal hydration level (CHL%) was measured to detect the ex vivo corneal damage after exposure to ITZ in CS/HA-DLs-T-G, CS-DLs-T-G, HA-DLs-T-G, DL-T-G, and ITZ-G (Table 3). The usual range of CHL is between 75 and 79%, which indicates no corneal damage [45]. The CHL% of all tested corneas was below 81%, suggesting no corneal damage during the ex vivo permeation study [19].

### 2.10. In Vitro Antifungal Activity

**Figure 7:**
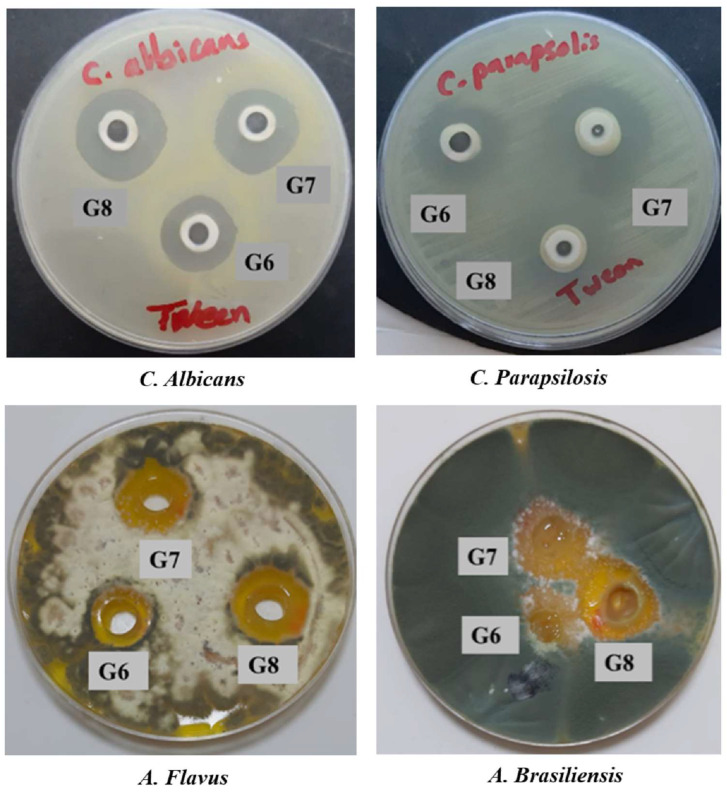
Antifungal activity profile of HA-ITZ-DLs-T-G (G6), CS-ITZ-DLs-T-G (G7), and CS/HA-ITZ-DLs-T-G (G6) using the cup plate method against different fungi.

The in vitro antifungal efficacy of HA-DLs-T-G, CS-DLs-T-G, and CS/HA-DLs-T-G was appraised using the agar-cup diffusion assay against two types of yeasts and molds, including *C. albicans*, *C. parapsilosis*, *A. flavus*, and *A. brasiliensis*. The inhibition zone results are presented in Table 4, with representative images of the agar plates (Figure 7 and Figure S3). All tested formulations exhibited antifungal activity (Figure 7) against the selected organisms compared to the control gel (C, Figure S3). This activity can be attributed to ITZ, which inhibits the cytochrome P-450-dependent enzyme lanosterol demethylase, which is essential for converting lanosterol to ergosterol [45,46]. Additionally, using TW80 contributed to a smaller size of DLs, potentially improving ITZ solubility and diffusion through the agar media. Among the formulations, ITZ-G showed the lowest antifungal activity (Figure S3), whereas CS/HA-DLs-T-G demonstrated the highest efficacy. The high antifungal effect of CS/HA-DLs-T-G can be ascribed to their double layer of CS and HA, which further supports its potent antifungal properties [13]. DLs coated with CS and HA can address antifungal resistance in *A. brasiliensis*. The primary mechanism of action is due to increased fungal cell membrane permeability, facilitated by the bioadhesive properties. The synergistic effects of DLs, CS, HA, and in situ gel provide a multifaceted strategy for efficient antifungal treatment [47]. The effect of CS and HA coatings in antifungal treatment is well-reported. The CS-coated nanoparticles showed notable antifungal efficacy. CS can enhance the penetration of fungal cell membranes due to its positive charge, improving the antifungal potency of the payload drugs [46]. HA displays fungistatic efficacy against different strains of *candida* [48]. The dual action of CS and HA in targeting fungal cell membranes supports their combined use in antifungal formulations. Senyiğit et al. developed an econazole nitrate-loaded CS-based in situ gel for vaginal candidiasis, signifying drug permeation with a high therapeutic effect [49]. The development in this area holds promise for effectively managing fungal infections using CS, HA-coated DLs–in situ gel formulations.

### 2.11. Corneal Retention Study

**Figure 8:**
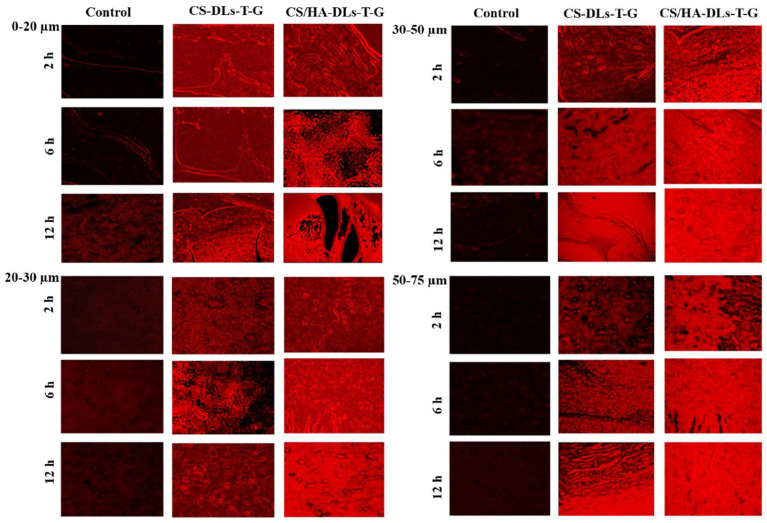
Florescent images of goat cornea after incubation with CS—DLs—T—G and CS/HA—DLs—T—G.

The retention experiment was further studied using fluorescence material (rhodamine B) to visualize their penetration across corneal layers. The depth of dye penetration was estimated under a fluorescence microscope at various thicknesses and time intervals, as illustrated in Figure 8.

CS-DLs-T-G and CS/HA-DLs-T-G were selected due to their excellent ocular ITZ permeation and antifungal activity. The rhodamine B solution exhibited limited penetration through the corneal tissue. In contrast, the obtained formulations showed significant retention throughout multiple depths of corneal tissues over time. A considerable intensity of fluorescent rhodamine B remained in the tissues after 6 and 12 h of treatment. Thus, the CS/HA-DLs-T-G formulation can effectively penetrate and prolong the retention of ITZ in the eye tissues. This behavior can be attributed to the combined effect of two biopolymers, HA and CS [48,50]. The remarkable retention capacity of CS/HA-DLs-T-G in the cornea presents significant advantages, such as extended drug delivery, improved bioavailability, and enhanced therapeutic efficacy of ophthalmic preparations. A limitation of this study is the utilization of an in vitro model, which does not consider physiological conditions such as blinking and tear drainage, impacting drug retention, and will be addressed in future in vivo studies.

### 2.12. Histology Study

**Figure 9:**
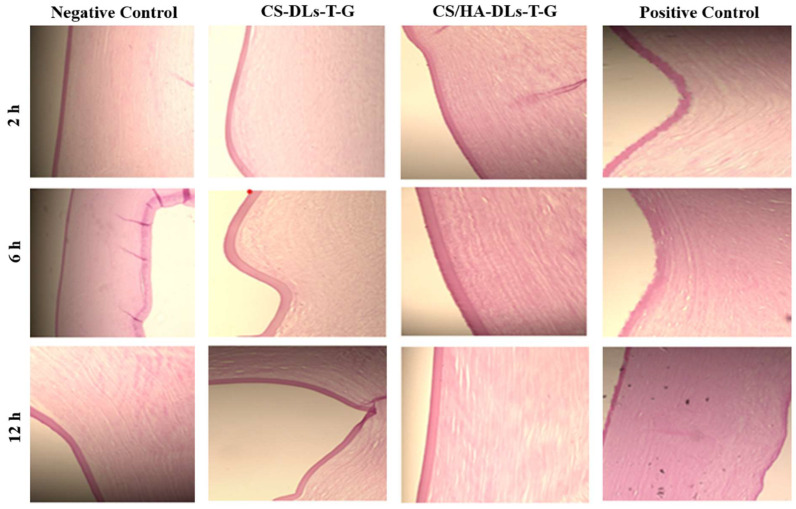
Cross-section images of the cornea after incubation with CS-DLs-T-G and CS/HA-DLs-T-G.

Figure 9 presents cross-section images of the cornea after 1, 6, and 12 h of exposure to CS-DLs-T-G and CS/HA-DLs-T-G. NaCl and KCl solutions were used as negative and positive controls, respectively. Marked damages and defects in the epithelium layer were observed in corneal tissues treated with a saturated solution of KCl [51].On the other hand, corneal tissues exposed to the formulations exhibited normal and intact epithelium and stroma layers. These indicate that the developed formulations are safe for ocular administration.

### 2.13. Ocular Irritation Study

**Figure 10:**
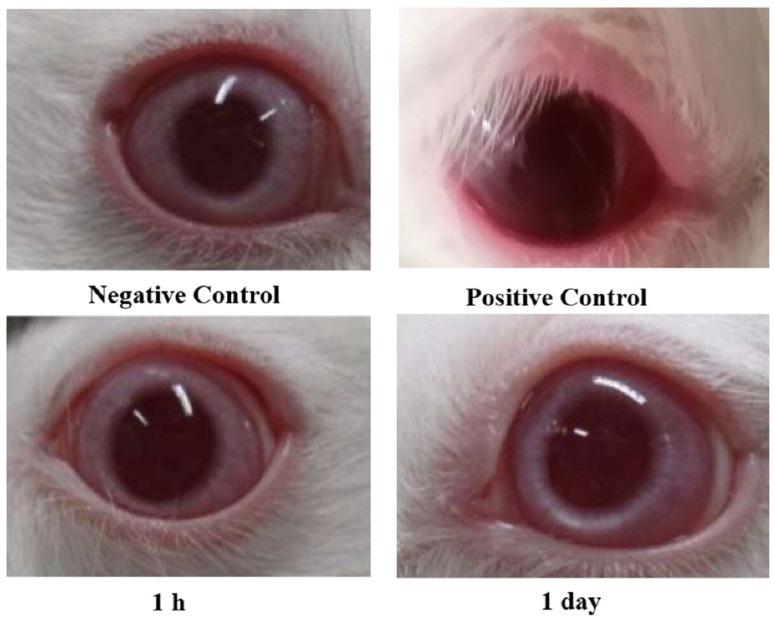
Ocular irritation testing on albino rabbits after application of CS/HA-DLs-T-G for 1 h and 1 day compared to KCL solution (positive control) and plain gel (negative control).

Plain DLs-T-G is considered a negative control and demonstrated no signs of irritation; in contrast, dioctyl sodium sulfosuccinate solution (positive control) caused significant irritation in the cornea, conjunctiva, and iris. Periodic observation of the rabbits after being treated with CS/HA-DLs-T-G revealed no eye redness, swelling, or watering (Table 5 and Figure 10). After 24 h, no traces of the formulations with non-irritation were observed, indicating complete clearance [31]. CS and HA are considered valuable biopolymers for ocular application due to they have natural hydrophilic, biodegradable, and mucoadhesive properties, while being non-irritating on eye tissues [52]. These biopolymer-coated nanocarriers can be engineered to enhance the drug’s ocular permeation, increasing pay-loaded drugs’ efficiency while minimizing ocular surface irritation. In vivo performance of formulation CS/HA-DLs-T-G showed formidable ocular tolerance after the administration into rabbit eyes. Complete preclinical studies are essential to assess the safety and effectiveness of these nanocarriers for ocular applications [53].

## 3. Conclusions

The ITZ-loaded DLs-T, coated with HA, CS, and bilayered CS/HA, were successfully optimized to achieve minimal vesicle size and maximal EE%. The optimized formulations exhibited nanoscaled vesicle sizes with spherical shapes. The coating led to a noticeable increase in vesicle sizes and a distinct change in zeta potential, confirming the successful formation of coating layers. These coatings effectively improved the EE% and provided sustained release profiles of ITZ. Additionally, the coated DLs-T were incorporated into pH-sensitive in situ gels, which showed appropriate gelling capacity, viscosity, and bioadhesion properties, confirming their suitability for an ocular application. Ex vivo studies demonstrated that the bilayer CS/HA-DLs-T-laden in situ gel improved ITZ transcorneal delivery and antifungal activity against various fungal strains. Future studies, like in vivo ocular delivery and efficacy investigation, are required to support our findings further. In conclusion, bilayer CS/HA-DLs-T-in situ gel is a practical approach to increase the ocular delivery of IT to treat ocular fungal infections.

## 4. Materials and Methods

### 4.1. Materials

Itraconazole (ITZ) was obtained from Betapharma (Shanghai, China), and soybean phosphatidylcholine (SPC, Lipoid S100, purity > 94%) was purchased from Lipoid GmBH (Ludwigshafen, Germany). Cholesterol (Chol) and low molecular weight chitosan (50–190 kDa) based on viscosity 20–300 cP were purchased from Sigma-Aldrich Co. LLC (St. Louis, MO, USA). Tween 80^®^ (TW80) was obtained from Koch-Light Laboratories Ltd. (Colnbrook Buck, UK). Poloxamer 188 (PL188) was obtained from Unichem Laboratories Ltd. (Mumbai, India). Hyaluronic acid sodium salt (HA; 1000 kDa) was obtained from Bloomage Freda Biopharm Co., Ltd. (Jinan, China). Acetonitrile, methanol, and ethanol HPLC grade were obtained from Fisher Scientific (Leicestershire, UK). Simulated tear fluid (STF) with a pH of 7.4 was prepared in our lab by dissolving 2 g of NaHCO_3_, 6.7 g of NaCl, and 0.8 g of CaCl_2_·2H_2_O in 1 L of water.

### 4.2. Preparation of ITZ Loaded Various Surface-Modified DLs

ITZ-loaded DLs were fabricated as previously described with minor modifications.

Briefly, SPC, Chol, and either TW 80 or PL188 were carefully combined in varying ratios to minimize the vesicle size and maximize entrapment efficiency (EE%). The initial ratio was 0.9%, 0.9%, and 0.1%, while another tested ratio was 0.9%, 0.1%, and 0.1% of SPC, TW80, and Chol, respectively. These components were melted in a glass tube at an elevated temperature (65 °C). In the adapted proliposomes–liposomes technique, ITZ and a few drops of ethanol were added to the molten lipid phase under stirring until all components were dissolved entirely. This mixture was then incorporated into an aqueous phase of preheated (65 °C) distilled water (200–400 µL) and allowed to cool to room temperature to obtain proliposome gel.

Different DLs were obtained by dispersing the proliposomes in 10 mL of PBS (pH 7.4) and stirring for 2 h at room temperature. DLs were incubated overnight at 4 °C to ensure complete swelling. Furthermore, different surface-modified DLs were successfully prepared, including HA-DLs, CS-DLs, and CS/HA double-layered DLs. HA-DLs and CS-DLs were formed after dispersing proliposomes in 10 mL of PBS (pH 7.4; 0.2% HA *w*/*v*) and 10 mL of CS solution (0.5% *w*/*v* dissolved in 0.1% *v*/*v* acetic acid), respectively. For CS/HA-DLs, proliposomes were first hydrated with 5 mL of 0.4% (*w*/*v*) HA solution and stirred for 2 h, followed by the addition of 5 mL of CS solution (1.0% *w*/*v*) with continuous stirring for another 2 h [8]. The formulations were then subjected to bath sonication for 1 h, followed by probe sonication for 10 min under an ice bath to produce nano-vesicles. The resulting formulations were centrifuged at 6000 rpm for 5 min to remove undissolved ITZ and large particles.

### 4.3. Characterization of ITZ-Loaded DLs

#### 4.3.1. Vesicle Size Distribution and Zeta Potential of ITZ-Loaded Biopolymer-Coated DLs

The vesicle size and polydispersity index (PdI) were detected via dynamic light scattering, while zeta potential values were assessed by electrophoretic mobility (Zetasizer Nano ZS, Malvern Instruments, Worcestershire, UK). The obtained formulations were diluted with Milli-Q water at 1:100 *v*/*v* at 25 °C with gentle vortexing. The presented values are the average of the three measurements.

#### 4.3.2. Entrapment Efficiency Measurement

Using an indirect technique, ITZ-loaded DLs were examined for entrapment efficiency (EE%). The unloaded ITZ (ITZ_F_, free drug) was separated from the prepared liposomes by ultracentrifugation for 30 min at 40,000 rpm and 4 °C. The amount of ITZ entrapped in DLs was calculated using the following equations:

(1) $$EE\%=\frac{{ITZ}_{T}-{ITZ}_{F}}{{ITZ}_{T}}\times 100$$

ITZ_T_ denotes the total amount of ITZ, which was determined by mixing 10 μL of each dispersion with 990 μL of acetonitrile to disrupt the vesicles and liberate ITZ. ITZ_F_ is the free ITZ, present in the supernatant after ultracentrifugation. A reversed-phase HPLC assay was used with minor modifications to measure the content of ITZ [45]. The mobile phase comprises deionized water and acetonitrile at a 30:70 (*v*/*v*) ratio, adjusted to pH 3.5 with glacial acetic acid. This mobile phase was pumped through a reversed-phase C_18_ column (PerkinElmer, 4.6 × 150 mm, 5 μm particle size) at a flow rate of 1.2 mL/min at room temperature. For each sample, an injection volume of 30 μL was used, and ITZ was detected using a UV detector at 245 nm. The HPLC system was controlled by software called “TotalChrom Workstation, version 6.3.1.”

#### 4.3.3. Stability Study

The obtained liposomal formulations were kept in tightly sealed amber-colored glass vials for one month at 4 °C. Samples were then taken periodically from each vial and investigated for their change in the vesicle size and zeta potential [7].

#### 4.3.4. Transmission Electron Microscopy

Transmission electron microscopy (TEM; JEM-2000EX II, JEOL, Tokyo, Japan) was used to visualize the morphology of the selected DLs. One drop of the selected DLs was applied to a copper grid, stained with a phosphotungstic acid solution (1% *w*/*v*), and left to dry at room temperature.

### 4.4. Preparation of ITZ-Loaded Biopolymer-Coated DLs–In Situ Gel

In situ gels are considered ideal carriers for ocular drug delivery due to their prolonged residence time and increased drug permeation. Therefore, the selected DLs (TW80), characterized by their smaller vesicle size and higher entrapment of ITZ, were incorporated into pH-triggered in situ gels. They were formulated using Carbopol 934P (0.2% *w*/*v*) and HPMC (0.8% *w*/*v*) after dispersing in 75% of the aqueous phase, followed by hydration and swelling overnight. The remaining 25% of the aqueous phase was then gradually added with continuous stirring until a uniform gel was formed. The gel dispersion also included benzalkonium chloride (0.02% *v*/*v*) as a preservative. A sufficient quantity of optimized DL pellets was collected after ultracentrifugation at 40,000 rpm for 15 min. ITZ content in the pellets was determined by dissolving them in 5 mL of methanol, followed by HPLC analysis. The prepared in situ gel was added to a measured weight of pellets to obtain 0.5% of ITZ under constant stirring. These formulations were designated DLs-T-G, HA-DLs-T-G, CS-DLs-T-G, and CS/HA-T-DLs-G. All preparation processes were conducted under aseptic conditions.

#### 4.4.1. Determination of pH, Gelation Time, and Rheology

The prepared in situ gels were characterized by pH, gelation time, and rheology. The pH of each formulation before and after mixing with STF was measured using a pH meter (Mettler Toledo, Greifensee, Switzerland), calibrated by standard buffers (pH 4 and 7). Each value represents the average of the three measurements. Gelation time is required for an in situ gel system to transition from a liquid to a gel state under physiological conditions. To simulate the ocular environment, 25 µL of the formulation was combined with 2 mL of simulated tear fluid (STF, pH 7.4) and maintained at 37 °C. The time needed for the gel to form was noticed by visual inspection [54]. The viscosity was measured by a DV-II+, Rotating Brookfield viscometer (Brookfield Engineering Laboratories Inc., Stoughton, MA, USA) using spindle No. 25 at shear rates ranging from 5 to 100 rpm. The viscosity measurement was performed at 25 °C without and with STF at pH 7.4 and 34 °C (physiological condition), as described by Sheshala et al. [55].

#### 4.4.2. Evaluation of Bioadhesion Proprieties

The bioadhesion characterization of ITZ-G, DLs-T-G, HA-DLs-T-G, CS-DLs-T-G, and CS/HA-T-DLs-G was appraised by measuring the impact of mucin on their zeta potential as an indicator of bioadhesion [7]. It was considered by measuring changes in the zeta potential of the formulation after incubation with negatively charged mucin. Accurately, 50 mg of each formulation was placed in 3 mL of mucin solution (0.5 mg/mL) at 35 °C for 1 h under stirring. The zeta potential values were then measured with a Zetasizer Nano ZS. The change in zeta potential values reflected the interaction between the formulations and mucin, which indicated their bioadhesive potential [7]. The experiment was conducted three times.

#### 4.4.3. In Vitro Release Study of ITZ-Loaded Biopolymer-Coated DLs–In Situ Gel

A dialysis membrane was employed to investigate the in vitro release profile of biopolymer-coated DLs–in situ gel formulation [56]. The STF (pH 7.4; 30 mL) containing 0.5% of TW80 was added to the beaker to preserve the sink condition. A cellulose membrane with a molecular weight cut-off of 8000–14,000 (Spectrum Laboratories Inc., Rancho Dominguez, CA, USA) was activated by soaking in distilled water overnight and then positioned at one end of the test tube. Equivalent weights of the formulation containing 5 mg of ITZ were separately filled in the test tube assembly, immersed in the release media (just the dialysis membrane), and rotated at 50 rpm at 37 °C using a water bath shaker. At predetermined interval times (1, 2, 4, 6, 8, 10, 12, and 24 h), a sample of 1 mL was taken from the baker and replaced by freshly prepared STF to confirm a constant volume. ITZ concentrations were determined using HPLC. Finally, ITZ’s release kinetics were calculated by fitting the obtained data to various kinetic models. The model with the highest correlation value (R^2^) best fits the ITZ release mechanism [57].

#### 4.4.4. Ex Vivo Corneal Permeation of ITZ-Loaded Biopolymer-Coated DLs–In Situ Gel

The transcorneal permeation study used freshly excised goat corneas in modified Franz cells with an exposed surface area of 2.5 cm^2^. Eyeballs were collected from a slaughterhouse and transported within 1 h in Ringer’s solution [5]. The corneas, with 3–5 mm of attached scleral tissues, were carefully dissected, rinsed with Ringer’s solution, and preserved in fresh STF, pH 7.4. Each excised cornea was mounted between the donor and receptor compartments at 37 ± 0.5 °C and 100 rpm. The receptor compartment was filled with STF (pH 7.4, 12.5 mL) containing 0.5% of Tween 80 to maintain sink conditions. The donor compartment was loaded with ITZ-DLs-Gels containing an equivalent of 5 mg of ITZ. The sample (1 mL) was periodically taken from the receptor compartment at intervals up to 8 h and replaced with an equivalent volume of fresh STF (pH 7.4). HPLC qualified the withdrawal solutions, representing the corneal permeated ITZ amount. The steady-state flux (*Jss*, [µg/(cm^2^ min)], transcorneal permeability coefficient (*P_eff_*, cm/min), and lag time (*T_L_*, h) were determined from the cumulative rate of transcorneal permeation. The experiment was repeated three times. The experiment was carried out according to the Guidelines of the Research Ethics Committee of King Saud University College of Pharmacy, Riyadh, Saudi Arabia (Ref. No.: KSU-SE-21-71).

#### 4.4.5. Corneal Hydration Level of ITZ-Loaded Biopolymer-Coated DLs–In Situ Gel

The goat corneas used for the ex vivo transcorneal permeation study were collected at the end of the test, weighed (W_1_), and dried in an oven at 90 °C [53]. These corneas were reweighed after drying (W_2_), and the percentage of corneal hydration (CH%) was calculated according to the following equation:

$$CH\%=\frac{{W1-W}_{2}}{W_{1}}\times 100$$

The corneal hydration level (CH) of 75–80% was considered normal; however, values above that limit were considered abnormal and reflected damage to epithelium and endothelium layers [5].

#### 4.4.6. In Vitro Antifungal Activity of ITZ-Loaded Biopolymer-Coated DLs–In Situ Gel

The antifungal activity of HA-DLs-T-G, CS-DLs-T-G, and CS/HA-DLs-T-G was performed due to their excellent ocular ITZ permeation. The in vitro antifungal efficacy was evaluated using the agar cup plate method against Candida albicans, Candida parapsilosis, Aspergillus flavus, and Aspergillus brasiliensis. These microorganisms were cultured in sterilized nutrient agar media on a Petri plate. Then, sterile wells with a 5 mm diameter were created using an s borer in each Petri dish. Therefore, 0.1 mL of each formulation was aseptically placed and labeled. The Petri plates were preserved at room temperature for 2 h to facilitate the diffusion of the formulations into the medium and then incubated at 28 °C for 48 h. The diameter of the inhibition zones was then measured in each well [51].

#### 4.4.7. Examination of Corneal Retention

Fluorescence microscopy was employed to assess the retention of CS-DLs-T-G and CS/HA-DLs-T-G in corneal tissue. Rhodamine B was used as a fluorescent substitute for ITZ to label the formulations. This allowed for the visualization of the corneal penetration at three different time intervals: 2, 6, and 12 h. The rhodamine B solution was used as a control. The excess fluorescent dye was removed from the corneal tissue and fixed in a 10% formic acid solution before fluorescence microscopy to minimize the background fluorescence. Fluorescence microscopy was conducted at an excitation wavelength of 540 nm and an emission wavelength of 625 nm [58]. This experiment was carried out according to the Guidelines of the Research Ethics Committee of King Saud University College of Pharmacy, Riyadh, Saudi Arabia (Ref. No.: KSU-SE-21-71).

#### 4.4.8. Histological Study

ITZ-DLs-Gels’ impact on the corneal tissues was histologically studied after 1, 6, and 12 h of incubation at 37 °C. The corneas were divided into four groups. Group 1 was incubated with the CS-DLs-T-G, and Group 2 received CS/HA-DLs-T-G. Group 3 was treated with PBS, pH 7.4 (negative control), while group 4 was incubated with a saturated KCl solution (positive control). The corneas were taken after 1, 6, and 12 h of incubation, washed with PBS (pH 7.4), and fixed with formalin solution (10% *v*/*v*) for 24 h. After that, the corneas were dehydrated with a subsequent series of ethanolic solutions, starting with 70% to 100% *v*/*v*, followed by Xylene. Then, the cornea was embedded in melted paraffin and left to cool down and solidify in blocks. Tissue sections less than 1 mm thick were sliced and placed on glass slides, stained with hematoxylin and eosin, and visualized under a microscope.

#### 4.4.9. Ocular Irritation Study

The Draize test was adapted to evaluate the irritation of ocular preparations by applying the tested formulation to the eye for appointed periods [57]. Compared to human eyes, rabbit eyes are more susceptible to irritating compounds. Score evaluation of the rabbit eye in modified Draize score was performed [7]. The ocular conditions were evaluated after 1 h and 1 day after applying 100 µL of CS/HA-DLs-T-G.

### 4.5. Statistical Data Analysis

Data analysis was conducted using the Microsoft Excel software package, Version 2010, and Origin software, Version 8. Results are expressed as mean ± deviation (n = 3). A one-way analysis of variance (ANOVA) was performed when comparing three or more conditions (*p* < 0.05).

## Figures and Tables

**Table 1:** Clarity, homogeneity, pH, and gelling capacity of ITZ-loaded biopolymer-coated DLs–in situ gel systems.

Formulations	Clarity	Homogeneity	pH		Gelling Capacity
			Without STF	With STF	
ITZ-G	Clear	Good	4.8	6.1	++
DLs-T-G	Clear	Good	5.5	6.7	++
HA-DLs-T-G	Clear	Good	5.5	6.7	++
CS-DLs-T-G	Clear	Good	4.8	6.5	+++
CS/HA-DLs-T-G	Clear	Good	4.9	6.6	+++

**Table 2:** In vitro release kinetics model of ITZ-loaded biopolymer-coated DLs–in situ gels.

Formulations	Zero Order	First Order	Hixon Crowell	Higuchi	Korsmeyer–Peppas	n Value
ITZ-G	0.9785	0.9836	0.9911	0.8768	0.9940	0.6182
DLs-T-G	0.9958	0.9856	0.9952	0.9022	0.9976	0.7044
HA-DLs-T-G	0.9845	0.9823	0.9845	0.9172	0.9909	0.7836
CS-DLs-T-G	0.9884	0.9739	0.9729	0.8874	0.9959	0.7991
CS/HA-DLs-T-G	0.9956	0.9847	0.9753	0.8996	0.9972	0.7573

**Table 3:** Steady-state flux (*Jss*), apparent permeability coefficient (*P_app_*), and CHL of ITZ-loaded biopolymer-coated DLs–in situ gels across goat cornea (n = 3).

Formulations	Jss [μg/(cm^2^·h)]	P_eff_ × 10^3^ (cm/h)	Lag Time	CHL
ITZ-G	18.84 ± 1.73	3.79 ± 0.35	1.76 ± 0.39	76.58 ± 1.12
DLs-T-G	45.73 ± 1.68	9.15 ± 0.15	0.57 ± 0.05	75.75 ± 0.69
HA-DLs-T-G	50.07 ± 1.20	10.01 ± 0.24	1.45 ± 0.12	75.18 ± 1.33
CS-DLs-T-G	59.54 ± 1.89	11.91 ± 017	1.58 ± 0.81	78.34 ± 0.71
CS/HA-DLs-T-G	66.13 ± 4.11	13.22 ± 0.82	1.25 ± 0.30	79.14 ± 1.01

**Table 4:** The antifungal activity level of HA-ITZ-DLs-T-G, CS-ITZ-DLs-T-G, and CS/HA-ITZ-DLs-T-G using the cup plate method against fungi and molds (inhibition zone in mm ± SD).

Formulations	Yeast		Mold	
	*C. albicans*	*C. parapsilosis*	*A. flavus*	*A. brasiliensis*
DLs-T-G	20.0 ± 0.02	24.0 ± 0.20	20.0 ± 0.06	16.0 ± 0.00
HA-DLs-T-G	16.0 ± 0.01	26.0 ± 0.02	18.0 ± 0.06	24.0 ± 0.00
CS-DLs-T-G	19.0 ± 0.00	24.0 ± 0.10	22.0 ± 0.03	26.0 ± 0.10
CS/HA-DLs-T-G	22.0 ± 0.10	28.0 ± 0.02	23.0 ± 0.00	28.0 ± 0.05

**Table 5:** Ocular irritation testing according to the Draize technique on albino rabbits (n = 3 per group).

	General Draize Score		
	Cornea (0–4)	Conjunctiva (0–3)	Iris (0–2)
CS/HA-DLs-T-G	0	0	0
Plain DLs-T-G (negative control)	0	0	0
Dioctyl sodium sulfosuccinate (positive control)	4	3	2

## Data Availability

Data are contained within the article.

## Supplementary Materials

The following supporting information can be downloaded at: https://www.mdpi.com/article/10.3390/gels11010019/s1, Figure S1: Stability study of ITZ-loaded biopolymer-coated DLs-T stored at 4 °C for 1-month duration based on the change of particle size (z-average), Figure S2: Stability study of ITZ-loaded biopolymer-coated DLs-T stored at 4 °C for 1-month duration based on the change of zeta potential, Figure S3: Antifungal activity profile of control gel (C), ITZ-G (G4), and DLs-T-G (G5) using cup plate method against different fungi.
