# Supplementary material for: Layer-by-Layer Biopolymer-Coated Deformable Liposomes–In Situ Gel: A Hybrid Strategy for Enhanced Ocular Delivery of Itraconazole: In Vitro and In Vivo Appraisal
Source: Gels. 2024 Dec 31;11(1):19. doi: 10.3390/gels11010019 (PMC11765087; doi:10.3390/gels11010019)
Supplement: Supplementary file 1 [file gels-11-00019-s001.zip › gels-3321919-supplementary.pdf]

Layer-by-Layer Biopolymers-Coated Deformable Liposomes-*In Situ* Gel: A Hybrid Strategy for Enhanced Ocular Delivery of Itraconazole: *In Vitro* and *In Vivo* Appraisal

Mohamed M. Badran [1,2*], Areej Alsubaie [1], Mounir M. Salem Bekhit [1], Abdullah H. Alomrani [1,2] and Aliyah Almomen [3]

[1] D Department of Pharmaceutics, College of Pharmacy, King Saud University, Riyadh 11495, Saudi Arabia; mbadran@ksu.edu.sa (M.M.B.); aomrani@ksu.edu.sa (A.H.A); mbekhet@KSU.EDU.SA; alalmomen@ksu.edu.sa (A.A)
[2] Nanobiotechnology Research Unit, College of Pharmacy, King Saud University, P.O. Box 2457, Riyadh 11495, Saudi Arabia
[3] Department of Pharmaceutical Chemistry, College of Pharmacy, King Saud University, Riyadh 11495, Saudi Arabia
* **Corresponding author: Mohamed M. Badran**, Department of Pharmaceutics, College of Pharmacy, King Saud University, Building # 23, AA 68, P.O. Box 2457, Riyadh 11451 Saudi Arabia; E-mail address: mbadran@ksu.edu.sa, mbadran75@gmail.com

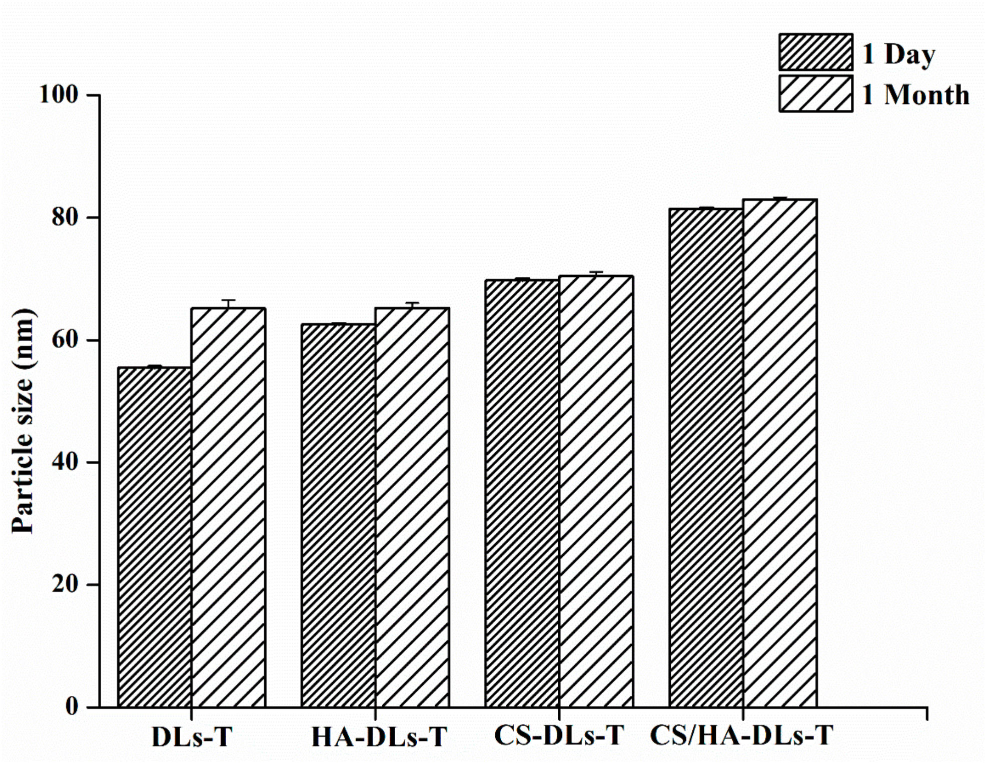

Figure S1. Stability study of ITZ-loaded biopolymer-coated DLs-T stored at 4°C for 1-month duration based on the change of particle size (z-average).

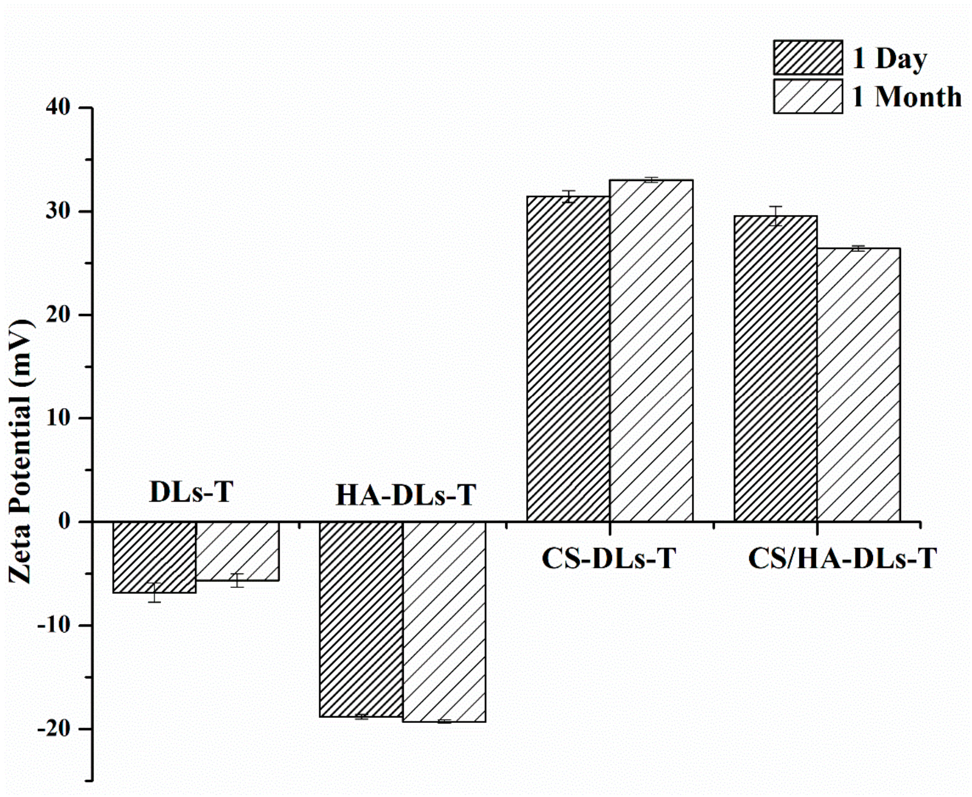

Figure S2. Stability study of ITZ-loaded biopolymer-coated DLs-T stored at 4°C for 1-month duration based on the change of zeta potential.

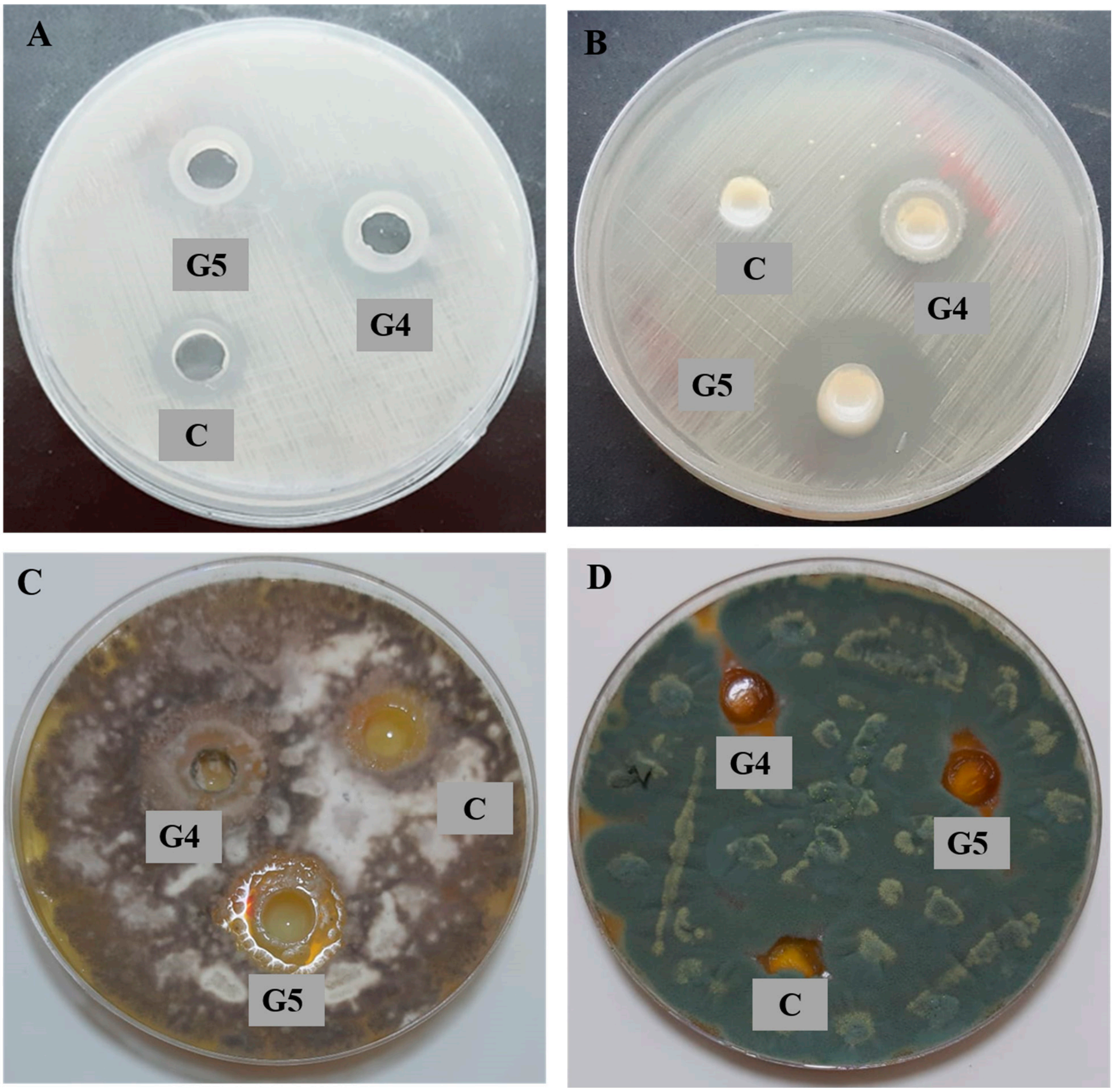

Figure S3. Antifungal activity profile of control gel (C), ITZ-G (G4), and DLs-T-G (G5) using cup plate method against different fungi.

(A)C. albicans, (B) C. parapsilosis, (C) A. flavus, and (D) A. brasiliensis.
